# Molecular Characterization of Clinical Linezolid-Resistant *Staphylococcus epidermidis* in a Tertiary Care Hospital

**DOI:** 10.3390/microorganisms11071805

**Published:** 2023-07-14

**Authors:** Florian Campmann, Hauke Tönnies, Christian Böing, Franziska Schuler, Alexander Mellmann, Vera Schwierzeck

**Affiliations:** 1 Institute of Hygiene, University Hospital Münster, 48149 Münster, Germany; florian.campmann@ukmuenster.de (F.C.); hauke.toennies@ukmuenster.de (H.T.); christian.boeing@ukmuenster.de (C.B.); alexander.mellmann@ukmuenster.de (A.M.); 2 Institute for Medical Microbiology, University Hospital Münster, 48149 Münster, Germany; franziska.schuler@ukmuenster.de

**Keywords:** *Staphylococcus epidermidis* ST2, linezolid resistance, WGS

## Abstract

*Staphylococcus epidermidis* (*S. epidermidis*) is part of the human skin flora but can also cause nosocomial infections, such as device-associated infections, especially in vulnerable patient groups. Here, we investigated clinical isolates of linezolid-resistant *S. epidermidis* (LRSE) collected from blood cultures at the University Hospital Münster (UHM) during the period 2020–2022. All detected isolates were subjected to whole genome sequencing (WGS) and the relatedness of the isolates was determined using core genome multilocus sequence typing (cgMLST). The 15 LRSE isolates detected were classified as multilocus sequence type (ST) 2 carrying the staphylococcal cassette chromosome *mec* (SCC*mec*) type III. All isolates showed high-level resistance for linezolid by gradient tests. However, no isolate carried the *cfr* gene that is often associated with linezolid resistance. Analysis of cgMLST data sets revealed a cluster of six closely related LRSE isolates, suggesting a transmission event on a hematological/oncological ward at our hospital. Among the included patients, the majority of patients affected by LRSE infections had underlying hematological malignancies. This confirms previous observations that this patient group is particularly vulnerable to LRSE infection. Our data emphasize that the surveillance of LRSE in the hospital setting is a necessary step to prevent the spread of multidrug-resistant *S. epidermidis* among vulnerable patient groups, such as patients with hematological malignancies, immunosuppression or patients in intensive care units.

## 1. Introduction

As a physical barrier and human body′s largest organ, the skin is physiologically colonized by a variety of different microorganisms, including *S. epidermidis.* Accordingly, the presence of *S. epidermidis* in clinical samples can often be interpreted as contamination or colonization. However, various studies [1,2,3] have shown that *S. epidermidis* can also play an important role in nosocomial infections. For this reason, physicians taking care of immunosuppressed patients or patients with hematological malignancies should be aware of the pathogenic property of *S. epidermidis* in regards to device-associated infections, such as catheter-related bloodstream infections. *S. epidermidis* has the ability to form a biofilm that impedes phagocytosis by immune cells and the effect of antimicrobial peptides, hence weakening the efficiency of antibiotic therapy [4]. In addition, device-associated infections cause enormous medical and economic burdens on healthcare systems worldwide [5].

In general, *S. epidermidis* displays resistance to various antimicrobial agents. A total of 60–70% of *S. epidermidis* isolates are resistant to commonly used first-line antibiotics including beta-lactam antibiotics. The most frequent resistance in *S. epidermidis* isolates is mediated by the *mecA* gene, which is encoded by a mobile genetic element called the staphylococcal cassette chromosome (SCCmec) and confers resistance to methicillin and other beta-lactam antibiotics [6].

Due to the widespread resistance to beta-lactam antibiotics, broad-spectrum antibiotics such as vancomycin are often prescribed in the treatment of serious *S. epidermidis* infections. In recent years, the oxazolidinone antibiotic linezolid, which is also used as a therapy for vancomycin-resistant enterococci (VRE) [2,7], has gained considerable importance in treating *S. epidermidis* infections. Due to its reduced nephrotoxicity in contrast to vancomycin, it is particularly suitable for severely ill and immunosuppressed patients.

Oxazolidinones disrupt bacterial protein synthesis by binding to the peptidyl transferase center of the ribosome, thereby inhibiting transition of the aminoacyl-tRNA to the A site. Until 2020, the global linezolid resistance rate in *S. epidermidis* was assumed to be low [7,8,9]. Nevertheless, various outbreaks and cases of nosocomial transmissions showed that LRSE strains are emerging as endemic pathogens in the hospital setting [8,9,10,11,12,13]. Severe illness, length of stay, and exposure to linezolid are cited as risk factors in several publications [2,8,9,10,12,14,15]. Most studies also cite overuse of linezolid during prolonged hospitalization as an additional risk factor. As postulated by R. Michels, it is suspected that coagulase negative *staphylococci* are predisposed to develop linezolid resistance after prolonged exposure to the antimicrobial agent [1,9,16,17,18]. So far, three different mechanisms of linezolid resistance have been identified. Firstly, the acquisition of the *cfr* gene located on plasmids, which codes for a ribosomal methyltransferase [19]; secondly, mutations in the 23S rRNA binding site of linezolid [17]; and finally, mutations in genes coding for the 50S ribosomal proteins L3, L4 and L22 of the peptide translocation center of the ribosome [8]. While the first published cases of linezolid-resistant isolates could be clearly attributed to spontaneous mutations, mostly affecting the 23S rRNA binding site and the 50S subunit of the ribosomal proteins, in the last two years, transmissible resistance mechanisms have emerged. These predominantly affect the *cfr* gene. Interestingly, the *cfr* gene was not only described in *staphylococci* and *enterococci*, but it could also be identified in other bacterial species such the *bacillus* species and even gram-negative bacteria. As Long et al. demonstrated in 2006 [20], the modification of A2503 in the *cfr* gene leads to a phenotype commonly referred to as PhLOPSA, which confers resistance to at least five antimicrobial classes (phenicols, lincosamides, oxazolidinones, pleuromutilins and streptogramin). Based on these effects, it is easy to understand why many publications have focused on the transmission and spread of *cfr* [21]. However, it remains to be seen to what extent *cfr* mutations are responsible for linezolid resistance in the clinical setting and hospital outbreaks [8,19,22,23].

The spread of LRSE in the hospital setting is a concern for infection control specialists as linezolid resistance restricts therapeutic treatment options to broad-spectrum antimicrobial agents. Furthermore, Coustilleres et al. illustrated in their study that LRSE bone and joint infections often require complex management as the patients tend to suffer from various comorbidities [13].

In this study, we investigated all LRSE isolates collected from blood cultures by whole genome sequencing (WGS) in the time period from 2020–2022 at the University Hospital Münster (UHM). Furthermore, we used WGS data to determine the clonal relationship between isolates, uncover potential transmission events and identify molecular resistance genes. We also evaluated the underlying diseases of the patients, as well as previously described risk factors such as their antibiotic pre-treatment, to determine which factors could contribute to linezolid resistance in our patient cohort.

## 2. Materials and Methods

### 2.1. Clinical Setting and Bacterial Isolates

The UHM is a 1500-bed tertiary care center admitting 55,000 patients per year in Münster, Germany. On average, 1539 positive blood cultures per year are detected at the UHM. Between January 2020 and December 2022, the centralized bacteriology laboratory of the UHM identified 15 LRSE isolates from different patients in blood cultures based on phenotypic linezolid susceptibility testing, as described below. Retrospectively, patient data in regards to date of blood culture sampling, patient age, attributed ward, duration of hospitalization on relevant ward and linezolid therapy were collected based on patients′ medical records and discharge letters. As sample collection and analysis were part of routine surveillance and infection control activities carried out in accordance with the national recommendations of the Robert-Koch Institute (Berlin, Germany), no formal patient consent was required for this study.

### 2.2. Microbiological Methods

At the Institute of Medical Microbiology, UHM, pairs of aerobic/anaerobic blood culture bottles (BD BACTEC TM plus Aerobic/F and BD BACTEC TM Plus Anaerobic/F, BD, Heidelberg, Germany) were incubated for five to seven days using an automated blood culture system (BD Bactec^TM^ FX Blood Culture System, Becton Dickinson, Heidelberg, Germany). Positive blood cultures were processed in accordance with German national guidelines [24]. First, positive blood cultures were microscopically examined after gram staining. Next, two drops of blood culture broth were spread on Columbia blood agar (Becton Dickinson, Heidelberg, Germany) and incubated at 5% CO_2_ and 36 ± 1 °C. Species were identified with matrix-assisted laser desorption ionization time-of-flight mass spectrometry (MALDI-TOF MS) (MALDI Microflex^®^ LT, Bruker, Bremen, Germany) using the reference Biotyper library v4.1 (Bruker Daltonik, Bremen, Germany). Antimicrobial susceptibility testing was performed using a Vitek2 automated system (bioMérieux, Marcy l′Étoile, France) applying EUCAST clinical breakpoints at the year of isolation, version 10.0 or 11.0, respectively. As a second step, linezolid resistance was confirmed using gradient tests (bioMérieux, Marcy l’Étoile, France) following EUCAST recommendations at the Institute of Hygiene, UHM. Results were interpreted according to EUCAST clinical breakpoints version 12.0.

### 2.3. Whole-Genome Sequencing and Data Analysis

All LRSE isolates were collected and cultured using standard procedures at the Institute of Hygiene, UHM. For DNA extraction, single colonies were picked, and genomic DNA (gDNA) of bacterial isolates was extracted using the NEB Monarch Genomic Purification Kit (New England Biolabs, Ipswich, MA, USA). For genomic comparison, all 15 LRSE isolates were subjected to WGS-based typing using the Pacific Biosciences Sequel IIe platform (Pacific Biosciences Inc., Menlo Park, CA, USA). All 15 sequences were submitted to BioProject (PRJNA988929). For this approach, we constructed the sequence library using the SMRTbell Express Template Prep Kit 2.0 in accordance with the manufacturer′s recommendations (Pacific Biosciences Inc.). The resulting reads were then assembled applying the “Microbial Assembly” pipeline within the SMRT Link software versions 10 and 11 (Pacific Biosciences Inc.) using default parameters. Only samples with ≥95% valid targets were analyzed further; otherwise, sequencing was repeated. For all WGS datasets, we utilized the software SeqSphere^+^ (Ridom GmbH, Münster, Germany, version 9.0.0) to compare coding regions in a gene-by-gene approach, i.e., core genome multilocus sequence typing (cgMLST), as described previously [25]. To analyze genetic relatedness, we used the recently published cgMLST scheme for *S. epidermidis* [26]. Clonal relationship of genotypes is here displayed using a minimum spanning tree algorithm calculated by the same software and is rated as closely related if genotypes differ in ≤6 alleles. MLST sequence types (STs) were extracted from the WGS data in silico. Linezolid and other key resistance genes were determined using the NCBI AMRFinderPlus [27] integrated in the SeqSphere^+^ software (version 9.0.0). To compare the genetic relatedness of the 15 LRSE isolates identified in our study with European isolates, we performed a literature search for ST2 LRSE isolates with available WGS data. All samples had to fulfill the following criteria: linezolid-resistant phenotype, clinical sample, ST2 genotype and high-quality WGS data set available. Only samples with ≥95% valid targets were included in our comparison. Afterwards, we used cgMLST to compare 15 isolates identified at the UHM to previously published LRSE samples [8,12,26,28]. For additional analysis, single nucleotide polymorphisms were extracted from core genome genes present in all isolates. A neighbor-joining was calculated with default parameters within the Ridom SeqSphere+ software and visualized using iTOL V. 6 [29].

### 2.4. Statistical Analysis

Descriptive statistics for categorical variables were provided by total number and percentage. In addition, means or medians were calculated where appropriate.

## 3. Results

### 3.1. Patients′ Characteristics

Among the 15 patients with LRSE identified in blood culture, 11 were male and 4 were female patients with a median age of 51 (21–92) years. The patients included were admitted to five different departments at our hospital, and their average length of hospital stay was 20 (5–35) days. Patients were exposed to multiple antibiotics for an average of 10 (4–16) days. Interestingly, many patients (n = 7) suffered from hematological malignancies, such as acute myeloid leukemia (AML), MDS-EB1 (MDS-EB1) and lymphoblastic T-cell lymphoma (T-LBL) (see Supplementary Table S1). Based on the clinical evaluation of their physicians, the detection of LRSE was considered an infection in six patients. This also corresponds to the fact that the majority of patients in our study cohort were considered vulnerable patients with multiple underlying co-morbidities or haemato-oncological malignancies. The only exception is patient P15 suffering from a cardiac disease. Interestingly, only P14 was treated with linezolid while the blood culture result was pending. 

### 3.2. Antimicrobial Susceptibility of LRSE Isolates

Phenotypic susceptibility testing of all LRSE isolates was performed using a Vitek2 automated system applying EUCAST clinical breakpoints at the year of isolation. Isolates were classified as susceptible (white), intermediate (grey) or resistant (black) according to the current EUCAST breakpoints (see Table 1). All LRSE isolates included in the study were resistant to methicillin as detected by phenotypic susceptibility testing. Furthermore, based on phenotypic antibiotic susceptibility testing, all isolates were susceptible to gentamycin, ciprofloxacin, levofloxacin, moxifloxacin, erythromycin, clindamycin, tetracycline and rifampicin. Only isolates P5, P9 and P11 were susceptible to trimethoprim; all other isolates showed resistance towards this antibiotic. On the other hand, all isolates were susceptible to the broad-spectrum antibiotics tigecycline, vancomycin and daptomycin. As a next step, we confirmed the phenotypic resistance for linezolid by gradient tests. Here, most isolates were classified as high-level linezolid-resistant with minimal inhibitory concentrations (MIC) of ≥48 mg/L [12]. The MIC of the isolates ranged between 32 and 256 mg/L.

### 3.3. Molecular Characterization of Isolates and Genotypic Relationship

WGS and subsequent extraction of the seven MLST targets identified all 15 LRSE isolates as ST2. This ST is frequently associated with nosocomial infections and blood stream infections [8,12,26,28]. In-depth analysis revealed that the SCC*mec* type III was detected in all isolates. Furthermore, all isolates carried the *mecA* gene, corresponding to the phenotypic antimicrobial susceptibility testing results. In addition, several other antimicrobial resistance genes were identified (see Table 2). WGS also shows that the *cfr* mutation was not found in any isolate. 

**Figure 1:**
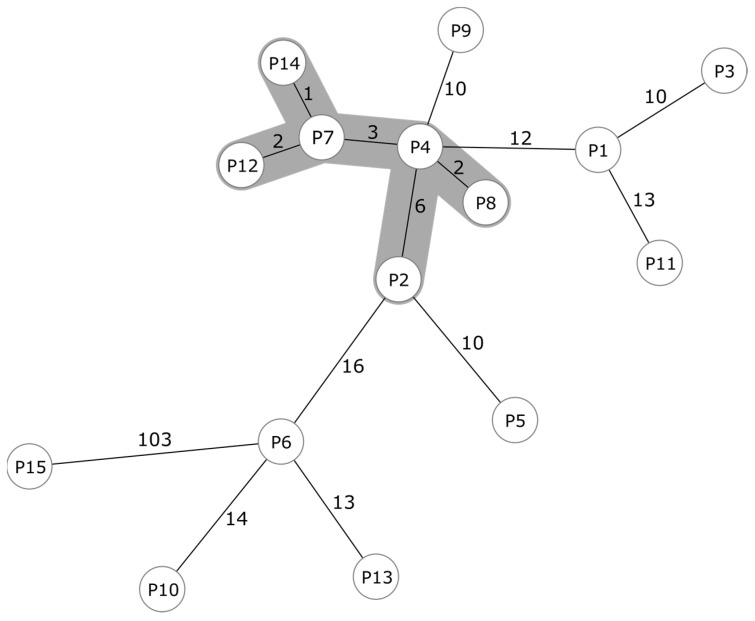
Minimum spanning tree of 15 LRSE isolates. The minimum spanning tree was based on up to 1846 cgMLST target genes, pairwise ignoring missing values. Every circle represents one genotype while the number on connecting lines represent the number of different alleles in a pairwise comparison. Isolates shaded in grey (P2, P4, P7, P8, P12, P14) differ in ≤6 alleles and are assumed to belong to a nosocomial cluster, indicating a transmission event on a hematological/oncological ward.

To determine the clonal relationship of the detected isolates, we extracted up to 1846 cgMLST targets from their WGS data and subsequently created a minimum spanning tree (Figure 1). Using the cluster threshold of ≤6 differing alleles, we detected a single cluster of six isolates (P2, P4, P7, P8, P12, P14) that differed in 1–6 alleles. Whereas five isolates (P4, P7, P8, P12, P14, depicted in grey) differed from each other in only ≤3 alleles, P2 was more distantly related with differences in six alleles. Indeed, epidemiological information revealed that the five corresponding patients could all be associated to one hematological/oncological ward. This observation further corroborated a clonal spread of LRSE. In contrast, the isolate P2 showed no epidemiological connection to the other cases. The remaining nine LRSE isolates were only distantly related to the potential outbreak cluster. These isolates showed 10 to 103 alleles difference to the described cluster. 

### 3.4. Comparison of European ST2 European Isolates Associated with the Hospital Setting

**Figure 2:**
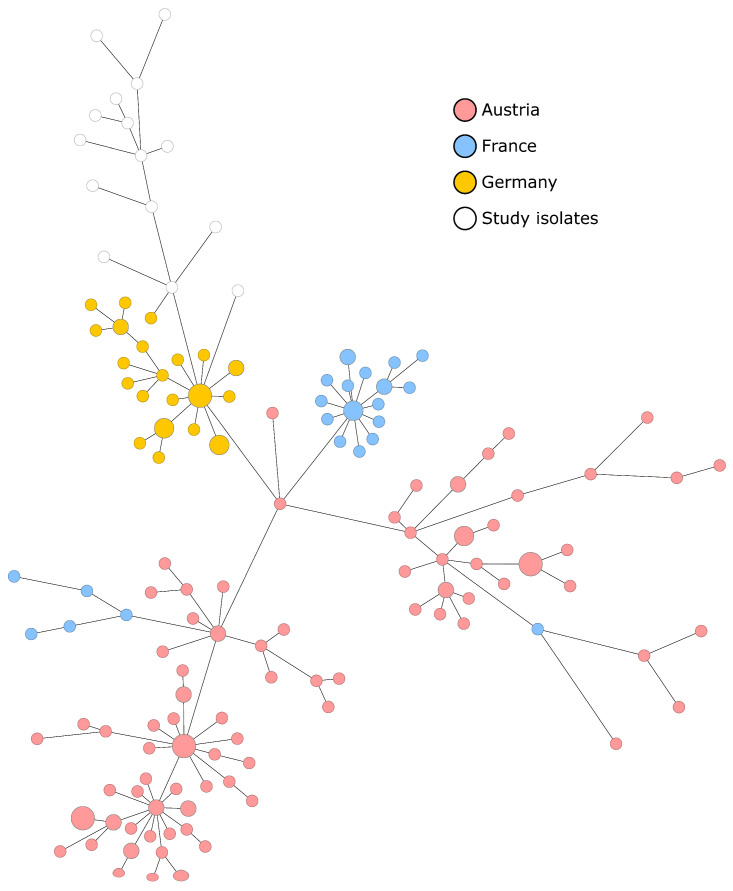
Minimum spanning tree comparing 175 European ST2 LRSE isolates. The analysis was based on up to 1846 cgMLST target genes, pairwise ignoring missing values. The length of connecting lines is proportional to the allelic differences. Circle sizes correspond to number of isolates, hence bigger circles indicate that several samples were genetically identical. The ST2 LRSE isolates were collected in hospitals located in France (n = 26), Germany (n = 31) and Austria (n = 103). The time of collection varied between 2011 and 2022. Isolates from our study (white) are genetically most similar to isolates collected at Saarland University Medical Center, a hospital located in Germany [26]. However, the allelic distance between isolates was at least 19 alleles.

ST2 has been shown to be among the most prevalent sequence types of *S. epidermidis* in hospitals [30]. As several publications have described nosocomial infections associated with ST2 LRSE, we wanted to investigate whether there are specific LRSE clones circulating in hospitals in Europe [8,12,26,31,32]. Using cgMLST, we compared our isolates with 160 European ST2 LRSE isolates described in the literature [8,12,26,28]. The isolates were collected in France (n = 26, collected in the years 2015–2018), Germany (n = 31, 2018–2020) and Austria (n = 103, 2011–2019). Based on cgMLST analysis, the isolates from our study appear most similar to the isolates collected in Germany but differ by at least 19 alleles (Figure 2). We also checked single nucleotide polymorphisms (SNPs) extracted from the core genome genes present in all isolates (Supplementary Table S2 and Figure S1). This approach also confirms the level of diversity among LRSE isolates in Europe. However, it has to be considered that the UHM and the hospital, where the other LRSE isolates were collected during the years 2018–2020 (the Saarland University Medical Center), are located approximately 290 kilometers apart in different federal states of Germany. Hence, hospital-to-hospital transmission is unlikely in this case, and our observations seem to represent strains that are circulating in the general population. Taken together, the genetic diversity of previously published European ST2 LRSE isolates makes the genetic relatedness of the identified transmission cluster more remarkable. It can also be interpreted as further evidence for a possible transmission connecting the samples of the described cluster.

## 4. Discussion

This study characterized all LRSE isolates collected from blood cultures over two years at the UHM by WGS. In total, 15 LRSE were identified during this time period. Gradient tests confirmed linezolid resistance for all isolates and indicated a high-level linezolid resistance with an average MIC of ≥48 mg/L. Genotyping based on WGS data classified the 15 isolates as ST2 with SCCmec type III conferring methicillin resistance (see Table 1). As the phenotypic resistance pattern of these isolates limits therapeutic treatment options to broad-spectrum antimicrobial agents, preventing further transmission of LRSE in the hospital setting is an important aim for infection control specialists. In fact, all patients in the study suffered from serious infections, such as bacteremia or device-associated blood stream infection, where treatment with an antimicrobial agent was vital.

Most isolates could be assigned to patients from a single haemato-oncology ward of our hospital (see Supplementary Table S1). WGS results and epidemiological investigation suggest the presence of a distinct clone on this ward (Figure 1). Indeed, 5 of 15 LRSE isolates shared closely related genotypes (P4, P7, P8, P12, P14) and differed among each other by less than three alleles. Interestingly, although 5 of 15 isolates very likely belonged to a single clone associated with the haemato-oncology ward, the isolate collected from patient P9, another patient that has stayed on this ward, differed by 10 alleles from this cluster (Figure 1). This could be a mere coincidence. Alternatively, if this is not a random occurrence, it could be explained by a time gap of approximately one year between the collection of isolates, which could have led to further differentiation and diversification of LRSE. On average, the five patients associated with the cluster had stayed on the oncology for three weeks. Patients P7, P12 and P14 had a simultaneous admission on the same ward for 8 days, making transmission likely. However, even in-depth epidemiological investigations could not identify any link for P2. Overall, based on WGS analysis, we identified a clonal transmission event that would not have been noticed without sequencing data.

Almost all patients in our study had underlying haemato-oncological diseases, among which AML was the most common (see Supplementary Table S1). Hence, as in previous studies, oncological diseases appeared to be a risk factor for the development of linezolid resistance in *S. epidermidis* in our patient cohort. While the causal relationship between linezolid usage and resistance has been demonstrated in most studies [7,23,33], in our study only a single patient (P14) received linezolid over a 7-day period prior to the LRSE positive blood culture. Mulanovich and colleagues postulated that exceeding a certain threshold of linezolid is necessary for selection pressure, i.e., for the occurrence of an outbreak [8,16,23]. However, in our case we conclude that the presence of LRSE on a haemato-oncological ward was rather caused by a transmission event than by selection pressure due to prolonged exposure to linezolid.

Another focus of this study is the absence of the *cfr* gene. Most new and older publications dealing with LRSE explicitly refer to resistance mediated by the *cfr* gene [6,10,11,15,16,17,22,23]. Other well-described mutations causing linezolid resistance are those in the 23S rRNA region, such as G2447T, T2500A and C2534T. Although the *cfr* gene was not identified in our samples, Table 1 shows that the MIC is relatively high for most isolates in our study. From this, we conclude that alternative mechanisms of resistance need to be considered. Furthermore, our results confirm previous research findings that ST2 LRSE spreads effectively in hospitals and, in particular, causes nosocomial bloodstream infections [10,17,21,24]. In addition, our study shows that LRSE isolates—even without *cfr* gene-carrying plasmids—can cause outbreaks in hospitals and pose a risk to vulnerable patient groups. Accordingly, further experimental studies and projects are necessary to identify and confirm the mutations that may cause the potential for high-level linezolid resistance.

Some limitations concerning our observations have to be taken into account: most importantly, the number of isolates collected in the last two years is small. Nevertheless, the importance of LRSE is underlined here, and the study gives further evidence that ST2 LRSE clones with a yet unknown linezolid-resistance mechanism are well adapted to the hospital setting. In general, LRSE isolates associated with nosocomial infections seem to show genetic variability, unless isolates can be traced to an outbreak (Figure 2). An analysis of previously published ST2 LRSE isolates showed no evidence of dominant LRSE clones circulating in different hospitals across Europe. However, the isolates in our study cohort are most similar to those identified in transmission events in Germany. Further studies will be needed to determine the local epidemiology of LRSE isolates in Europe.

## 5. Conclusions

WGS data analysis revealed a transmission event of ST2 LRSE on a hematological/oncological ward at our hospital. All isolates showed high-level resistance for linezolid in phenotypic susceptibility testing. Considering that all patients in our study were immunosuppressed, we assume that this vulnerable group of patients will benefit from strategies to prevent further transmission of LRSE strains in the hospital setting.

## Figures and Tables

**Table 1:** Phenotypic antimicrobial resistance patterns in LRSE isolates.

Isolate	OXA	GEN	CIP	LEV	MXF	ERY	CL	TGC	DAP	VAN	TET	RIF	TMP	LZD	LZD (MIC mg/L)
P1	R	R	R	R	R	I	R	S	S	S	R	R	R	R	>256
P2	R	R	R	R	R	R	R	S	S	S	R	R	R	R	>32
P3	R	R	R	R	R	R	R	S	S	S	R	R	R	R	>256
P4	R	R	R	R	R	R	R	S	S	S	R	R	R	R	>256
P5	R	R	R	R	R	R	R	S	S	S	R	R	S	R	>32
P6	R	R	R	R	R	R	R	S	S	S	R	R	R	R	>32
P7	R	R	R	R	R	R	R	S	S	S	R	R	R	R	>256
P8	R	R	R	R	R	R	R	S	S	S	R	R	R	R	>256
P9	R	R	R	R	R	R	R	S	S	S	R	R	S	R	>256
P10	R	R	R	R	R	R	R	S	S	S	R	R	R	R	>64
P11	R	R	R	R	R	R	R	S	S	S	R	R	S	R	>128
P12	R	R	R	R	R	R	R	S	S	S	R	R	R	R	>64
P13	R	R	R	R	R	R	R	S	S	S	R	R	R	R	>256
P14	R	R	R	R	R	R	R	S	S	S	R	R	R	R	>32
P15	R	R	R	R	R	R	R	S	S	S	R	R	R	R	>64

Abbreviations: OXA: oxacillin; GEN: gentamycin; CIP: cipofloxacin; LEV: levofloxacin; MXF: moxifloxacin; ERY: erytromycin; CL: clindamycin; DAP: daptomycin; VAN: vancomycin; TET: tetracyclin; RIF: rifampicin; TMP: trimethoprim; TGC: tigecyclin; LZD: linezolid; MIC: minimal inhibitory concentration; color map: black: resistant, grey: intermediate, white: susceptible.

**Table 2:** Antimicrobial resistance genes in LRSE isolates.

Isolate	AK	GEN	KA	T	BLA	MC	F	FA	TMP
P11	*aac(6′)-Ie/aph(2″)-Ia*	*aac(6′)-Ie/aph(2″)-Ia*	*aac(6′)-Ie/aph(2″)-Ia*	*aac(6′)-Ie/aph(2″)-Ia*	*blaZ*	*mecA mecI mecR1*	*fosB*	*fusB*	*dfrS1*
P9	*aac(6′)-Ie/aph(2″)-Ia*	*aac(6′)-Ie/aph(2″)-Ia*	*aac(6′)-Ie/aph(2″)-Ia*	*aac(6′)-Ie/aph(2″)-Ia*	*blaI blaZ*	*mecA mecI mecR1*	*fosB*	*fusB*	*dfrS1*
P15	*aac(6′)-Ie/aph(2″)-Ia*	*aac(6′)-Ie/aph(2″)-Ia*	*aac(6′)-Ie/aph(2″)-Ia*	*aac(6′)-Ie/aph(2″)-Ia*	*blaI blaZ*	*mecA mecI mecR1*	*fosB*	*fusB*	*dfrS1*
P10	*aac(6′)-Ie/aph(2″)-Ia*	*aac(6′)-Ie/aph(2″)-Ia*	*aac(6′)-Ie/aph(2″)-Ia*	*aac(6′)-Ie/aph(2″)-Ia*	*blaI blaZ*	*mecA mecI mecR1*	*fosB*	*fusB*	*dfrS1*
P12	*aac(6′)-Ie/aph(2″)-Ia*	*aac(6′)-Ie/aph(2″)-Ia*	*aac(6′)-Ie/aph(2″)-Ia*	*aac(6′)-Ie/aph(2″)-Ia*	*blaI blaZ*	*mecA mecI mecR1*	*fosB*	*fusB*	*dfrS1*
P13	*aac(6′)-Ie/aph(2″)-Ia*	*aac(6′)-Ie/aph(2″)-Ia*	*aac(6′)-Ie/aph(2″)-Ia*	*aac(6′)-Ie/aph(2″)-Ia*	*blaI blaZ*	*mecA mecI mecR1*	*fosB*	*fusB*	*dfrS1*
P5	*aac(6′)-Ie/aph(2″)-Ia*	*aac(6′)-Ie/aph(2″)-Ia*	*aac(6′)-Ie/aph(2″)-Ia*	*aac(6′)-Ie/aph(2″)-Ia*	*blaI blaZ*	*mecA mecI mecR1*	*fosB*	*fusB*	*dfrS1*
P14	*aac(6′)-Ie/aph(2″)-Ia*	*aac(6′)-Ie/aph(2″)-Ia*	*aac(6′)-Ie/aph(2″)-Ia*	*aac(6′)-Ie/aph(2″)-Ia*	*blaI blaZ*	*mecA mecI mecR1*	*fosB*	*fusB*	*dfrS1*
P2	*aph(3′)-IIIa*		*aph(3′)-IIIa*		*blaI blaZ*	*mecA mecI mecR1*	*fosB*	*fusB*	*dfrS1*
P4	*aac(6′)-Ie/aph(2″)-Ia*	*aac(6′)-Ie/aph(2″)-Ia*	*aac(6′)-Ie/aph(2″)-Ia*	*aac(6′)-Ie/aph(2″)-Ia*	*blaI blaZ*	*mecA mecI mecR1*	*fosB*	*fusB*	*dfrS1*
P1	*aac(6′)-Ie/aph(2″)-Ia*	*aac(6′)-Ie/aph(2″)-Ia*	*aac(6′)-Ie/aph(2″)-Ia*	*aac(6′)-Ie/aph(2″)-Ia*	*blaI blaZ*	*mecA mecI mecR1*	*fusB*	*fusB*	*dfrS1*
P6	*aac(6′)-Ie/aph(2″)-Ia*	*aac(6′)-Ie/aph(2″)-Ia*	*aac(6′)-Ie/aph(2″)-Ia*	*aac(6′)-Ie/aph(2″)-Ia*	*blaI blaZ*	*mecA mecI mecR1*	*fosB*	*fusB*	*dfrS1*
P7	*aac(6′)-Ie/aph(2″)-Ia*	*aac(6′)-Ie/aph(2″)-Ia*	*aac(6′)-Ie/aph(2″)-Ia*	*aac(6′)-Ie/aph(2″)-Ia*	*blaI blaZ*	*mecA mecI mecR1*	*fosB*	*fusB*	*dfrS1*
P3	*aac(6′)-Ie/aph(2″)-Ia*	*aac(6′)-Ie/aph(2″)-Ia*	*aac(6′)-Ie/aph(2″)-Ia*	*aac(6′)-Ie/aph(2″)-Ia*	*blaI blaZ*	*mecA mecI mecR1*	*fosB*	*fusB*	*dfrS1*
P8	*aac(6′)-Ie/aph(2″)-Ia*	*aac(6′)-Ie/aph(2″)-Ia*	*aac(6′)-Ie/aph(2″)-Ia*	*aac(6′)-Ie/aph(2″)-Ia*	*blaI blaZ*	*mecA mecI mecR1*	*fosB*	*fusB*	*dfrS1*

Abbreviations: AK: amikacin; GEN: gentamycin; KA: kanamycin; T: tobramycin; BLA: beta-lactam; F: fosfomycin; MC: methicillin; FA: fusidic acid; TMP: trimethoprim.

## Data Availability

Sequences of all 15 LRSE isolates were submitted to BioProject (PRJNA988929) prior to publication.

## Supplementary Materials

The following supporting information can be downloaded at https://www.mdpi.com/article/10.3390/microorganisms11071805/s1: Figure S1: Neighbor-joining (NJ) tree of 175 *S. epidermidis* ST2 global isolates; Table S1: Characteristics of patients included in the study; Table S2: List of 175 *S. epidermidis* isolates used for comparison.
