# Supplementary material for: Molecular Characterization of Clinical Linezolid-Resistant Staphylococcus epidermidis in a Tertiary Care Hospital
Source: Microorganisms. 2023 Jul 14;11(7):1805. doi: 10.3390/microorganisms11071805 (PMC10383320; doi:10.3390/microorganisms11071805)
Supplement: Supplementary file 1 [file microorganisms-11-01805-s001.zip › microorganisms-2474640-supplementary/supplementary table S1 and figure S1.pdf]

**Supplementary Table S1.** Characteristics of patients included in the study

| Patient | Date of culture | Clinical Department | Sex | Previous therapy with linezolid? | underlying medical condition |
|---|---|---|---|---|---|
| P1 | 24/01/2020 | gastro-enterology | female | no | toxic liver cirrhosis |
| P2 | 09/04/2020 | gastro-enterology | male | no | liver graft failure |
| P3 | 10/06/2020 | gastro-enterology | male | no | toxic liver cirrhosis |
| P4 | 01/07/2020 | hematology | male | no | AML |
| P5 | 07/07/2020 | ICU | male | no | liver failure/HCC |
| P6 | 14/08/2020 | hematology | female | no | NASH/liver failure |
| P7 | 02/02/2021 | hematology | male | no | AML |
| P8 | 22/02/2021 | hematology | male | yes | T-LBL |
| P9 | 06/06/2021 | hematology | female | no | AML |
| P10 | 05/07/2021 | hematology | male | no | AML |
| P11 | 23/07/2021 | hematology | male | no | MDS-EB1 |
| P12 | 12/08/2021 | hematology | female | no | NASH/liver failure |
| P13 | 12/08/2021 | ICU | male | no | Crohn's disease |
| P14 | 29/08/2021 | hematology | male | no | AML |
| P15 | 02/09/2021 | infectious disease | male | no | chronic heart failure |

Abbreviations: ICU: Intensive care unit; AML: Acute myeloid leukemia; HCC: hepatocellular carcinoma; T-LBL: Lymphoblastic T-cell lymphoma; MDS-EB1: Myelodysplastic syndrome EB1; NASH: non-alcoholic steatohepatitis.

**Supplementary Figure S1**

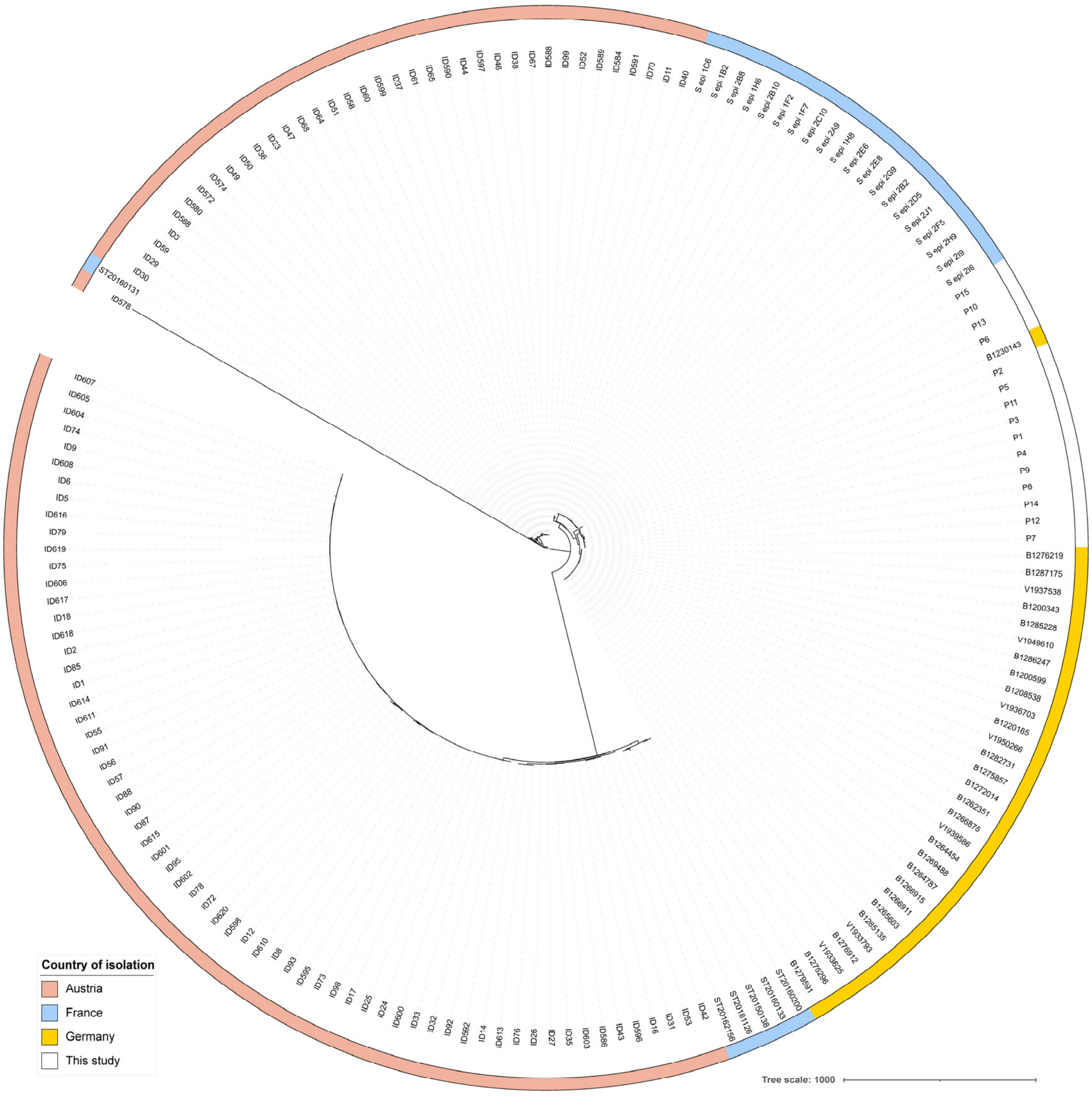


**Supplementary Figure S1: Neighbor-joining (NJ) tree of 175 S. epidermidis ST2 global isolates.** Single nucleotide polymorphisms (n = 4,328) were extracted from 1,502 core genome genes present in all isolates and formed the basis to calculate the NJ tree with default parameters within the Ridom SeqSphere+ software. We used iTOL V. 6 [29] to display the tree and metadata of the strains. The leaves of the tree were annotated with the sample names. The colored circle indicates the country of isolation.
